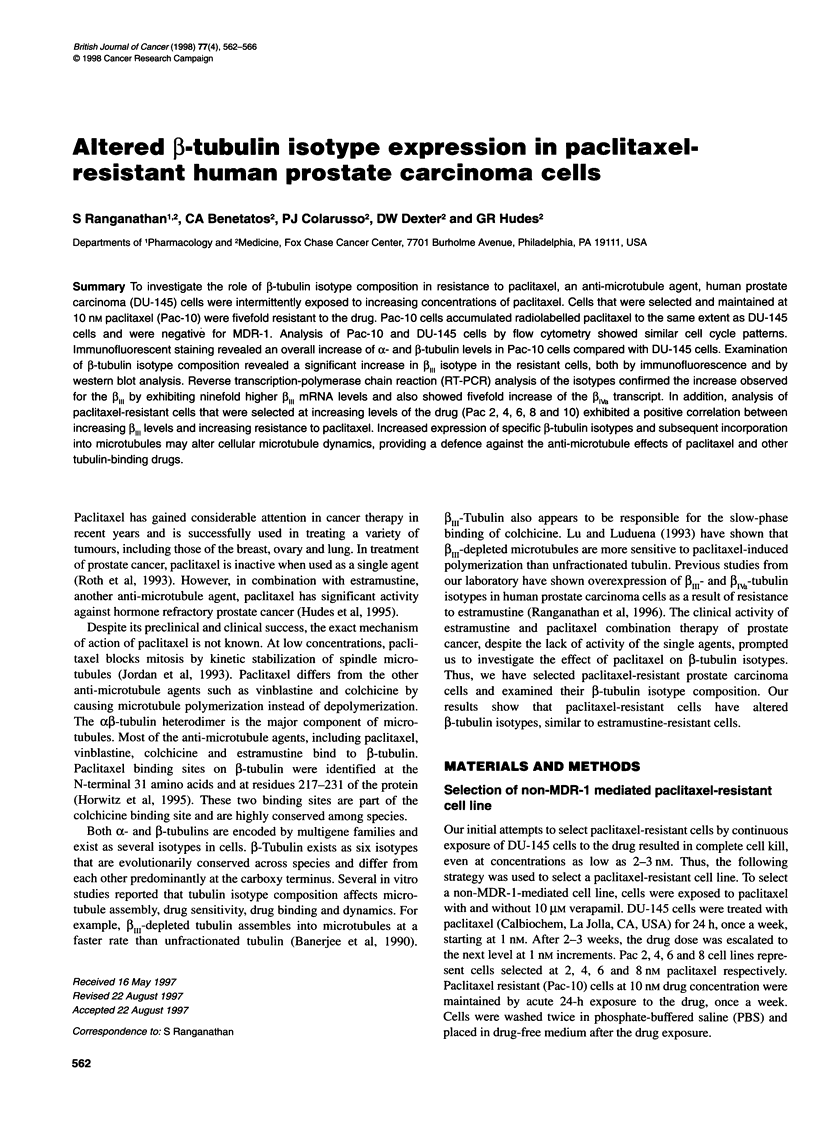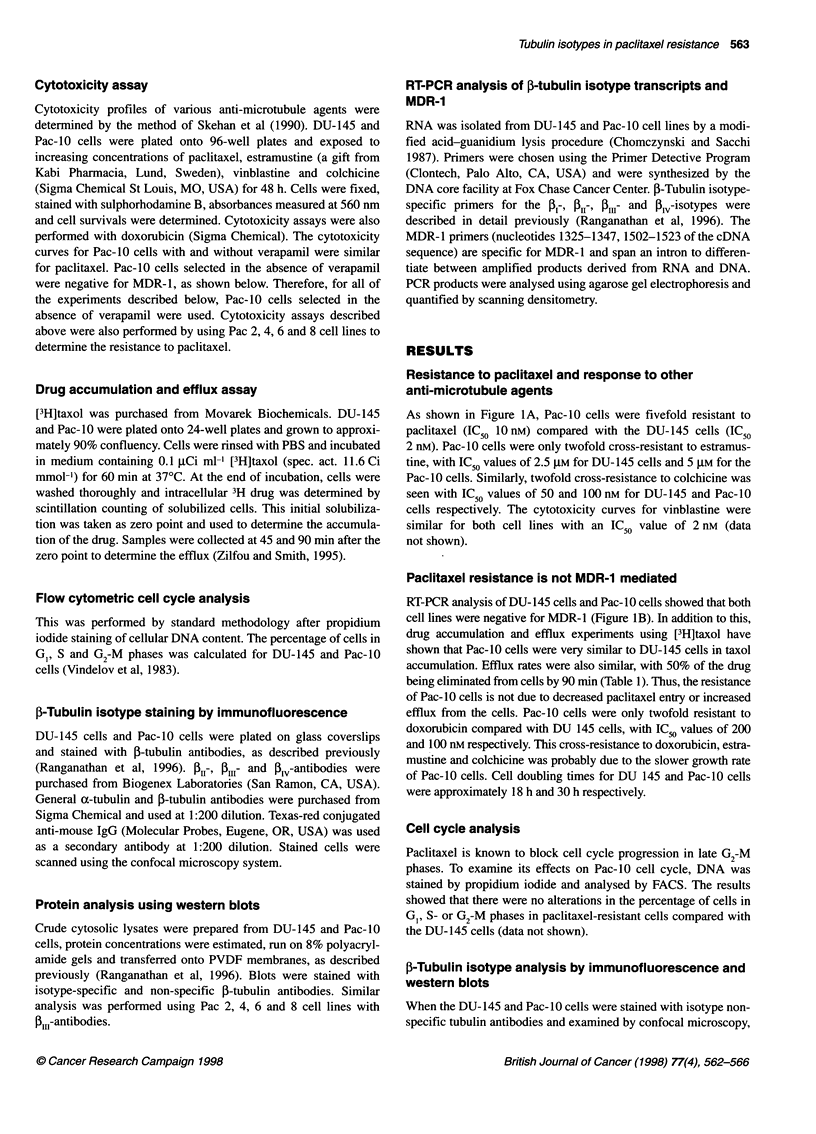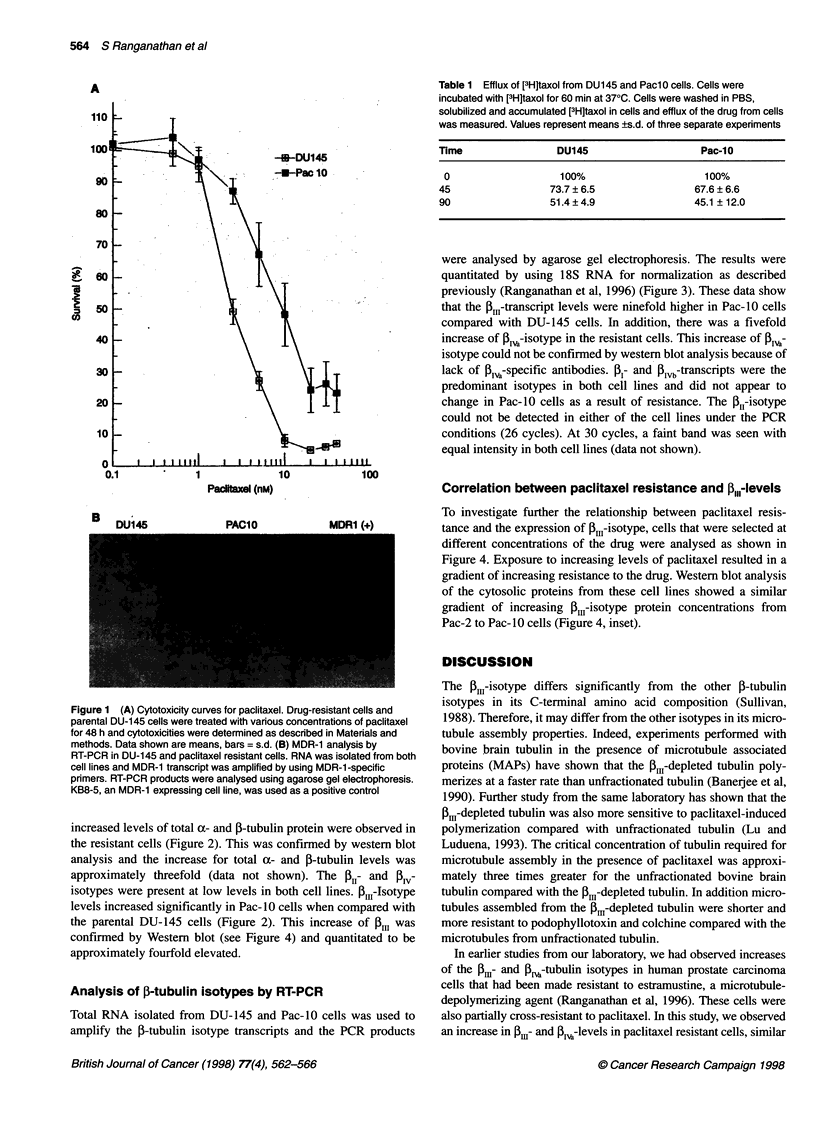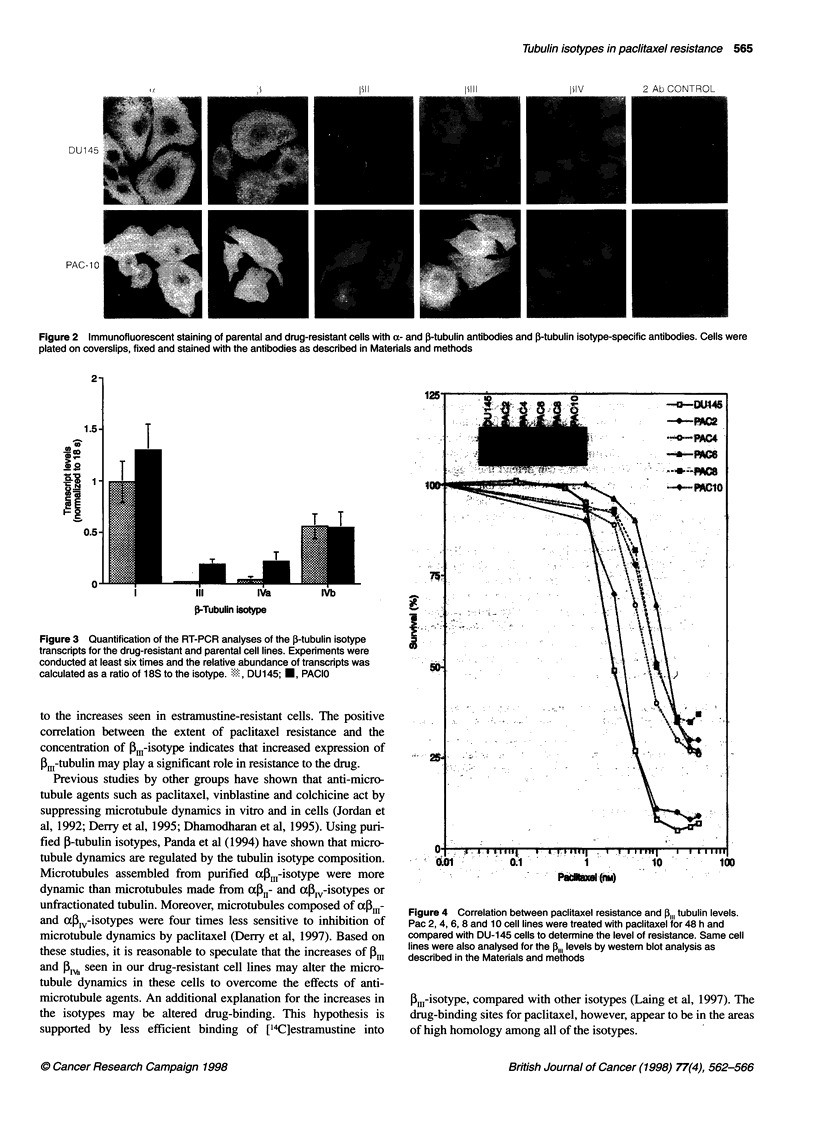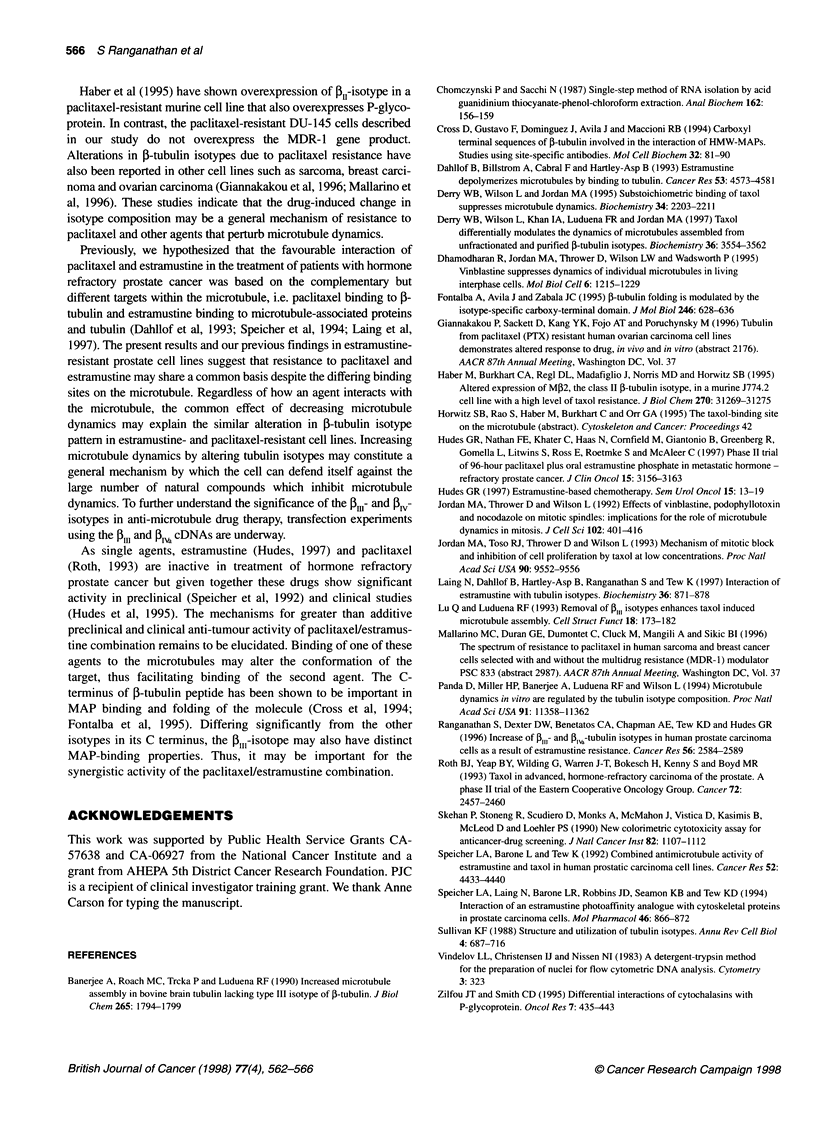# Altered beta-tubulin isotype expression in paclitaxel-resistant human prostate carcinoma cells.

**DOI:** 10.1038/bjc.1998.91

**Published:** 1998-02

**Authors:** S. Ranganathan, C. A. Benetatos, P. J. Colarusso, D. W. Dexter, G. R. Hudes

**Affiliations:** Department of Pharmacology, Fox Chase Cancer Center, Philadelphia, PA 19111, USA.

## Abstract

**Images:**


British Journal of Cancer (1998) 77(4), 562–566
© 1998 Cancer Research Campaign

### Altered β-tubulin isotype expression in paclitaxel-resistant human prostate carcinoma cells

S Ranganathan[1,2], CA Benetatos[2], PJ Colarusso[2], DW Dexter[2] and GR Hudes[2]

Departments of [1]Pharmacology and [2]Medicine, Fox Chase Cancer Center, 7701 Burholme Avenue, Philadelphia, PA 19111, USA

**Summary** To investigate the role of β-tubulin isotype composition in resistance to paclitaxel, an anti-microtubule agent, human prostate carcinoma (DU-145) cells were intermittently exposed to increasing concentrations of paclitaxel. Cells that were selected and maintained at 10 nM paclitaxel (Pac-10) were fivefold resistant to the drug. Pac-10 cells accumulated radiolabelled paclitaxel to the same extent as DU-145 cells and were negative for MDR-1. Analysis of Pac-10 and DU-145 cells by flow cytometry showed similar cell cycle patterns. Immunofluorescent staining revealed an overall increase of α- and β-tubulin levels in Pac-10 cells compared with DU-145 cells. Examination of β-tubulin isotype composition revealed a significant increase in $\beta_{III}$ isotype in the resistant cells, both by immunofluorescence and by western blot analysis. Reverse transcription-polymerase chain reaction (RT-PCR) analysis of the isotypes confirmed the increase observed for the $\beta_{III}$ by exhibiting ninefold higher $\beta_{III}$ mRNA levels and also showed fivefold increase of the $\beta_{IVa}$ transcript. In addition, analysis of paclitaxel-resistant cells that were selected at increasing levels of the drug (Pac 2, 4, 6, 8 and 10) exhibited a positive correlation between increasing $\beta_{III}$ levels and increasing resistance to paclitaxel. Increased expression of specific β-tubulin isotypes and subsequent incorporation into microtubules may alter cellular microtubule dynamics, providing a defence against the anti-microtubule effects of paclitaxel and other tubulin-binding drugs.

Paclitaxel has gained considerable attention in cancer therapy in recent years and is successfully used in treating a variety of tumours, including those of the breast, ovary and lung. In treatment of prostate cancer, paclitaxel is inactive when used as a single agent (Roth et al, 1993). However, in combination with estramustine, another anti-microtubule agent, paclitaxel has significant activity against hormone refractory prostate cancer (Hudes et al, 1995).

Despite its preclinical and clinical success, the exact mechanism of action of paclitaxel is not known. At low concentrations, paclitaxel blocks mitosis by kinetic stabilization of spindle microtubules (Jordan et al, 1993). Paclitaxel differs from the other anti-microtubule agents such as vinblastine and colchicine by causing microtubule polymerization instead of depolymerization. The αβ-tubulin heterodimer is the major component of microtubules. Most of the anti-microtubule agents, including paclitaxel, vinblastine, colchicine and estramustine bind to β-tubulin. Paclitaxel binding sites on β-tubulin were identified at the N-terminal 31 amino acids and at residues 217–231 of the protein (Horwitz et al, 1995). These two binding sites are part of the colchicine binding site and are highly conserved among species.

Both α- and β-tubulins are encoded by multigene families and exist as several isotypes in cells. β-Tubulin exists as six isotypes that are evolutionarily conserved across species and differ from each other predominantly at the carboxy terminus. Several in vitro studies reported that tubulin isotype composition affects microtubule assembly, drug sensitivity, drug binding and dynamics. For example, $\beta_{III}$-depleted tubulin assembles into microtubules at a faster rate than unfractionated tubulin (Banerjee et al, 1990).

*Received 16 May 1997*
*Revised 22 August 1997*
*Accepted 22 August 1997*

*Correspondence to:* S Ranganathan

$\beta_{III}$-Tubulin also appears to be responsible for the slow-phase binding of colchicine. Lu and Luduena (1993) have shown that $\beta_{III}$-depleted microtubules are more sensitive to paclitaxel-induced polymerization than unfractionated tubulin. Previous studies from our laboratory have shown overexpression of $\beta_{III}$- and $\beta_{IVa}$-tubulin isotypes in human prostate carcinoma cells as a result of resistance to estramustine (Ranganathan et al, 1996). The clinical activity of estramustine and paclitaxel combination therapy of prostate cancer, despite the lack of activity of the single agents, prompted us to investigate the effect of paclitaxel on β-tubulin isotypes. Thus, we have selected paclitaxel-resistant prostate carcinoma cells and examined their β-tubulin isotype composition. Our results show that paclitaxel-resistant cells have altered β-tubulin isotypes, similar to estramustine-resistant cells.

### MATERIALS AND METHODS

### Selection of non-MDR-1 mediated paclitaxel-resistant cell line

Our initial attempts to select paclitaxel-resistant cells by continuous exposure of DU-145 cells to the drug resulted in complete cell kill, even at concentrations as low as 2–3 nM. Thus, the following strategy was used to select a paclitaxel-resistant cell line. To select a non-MDR-1-mediated cell line, cells were exposed to paclitaxel with and without 10 μM verapamil. DU-145 cells were treated with paclitaxel (Calbiochem, La Jolla, CA, USA) for 24 h, once a week, starting at 1 nM. After 2–3 weeks, the drug dose was escalated to the next level at 1 nM increments. Pac 2, 4, 6 and 8 cell lines represent cells selected at 2, 4, 6 and 8 nM paclitaxel respectively. Paclitaxel resistant (Pac-10) cells at 10 nM drug concentration were maintained by acute 24-h exposure to the drug, once a week. Cells were washed twice in phosphate-buffered saline (PBS) and placed in drug-free medium after the drug exposure.

### Cytotoxicity assay

Cytotoxicity profiles of various anti-microtubule agents were determined by the method of Skehan et al (1990). DU-145 and Pac-10 cells were plated onto 96-well plates and exposed to increasing concentrations of paclitaxel, estramustine (a gift from Kabi Pharmacia, Lund, Sweden), vinblastine and colchicine (Sigma Chemical St Louis, MO, USA) for 48 h. Cells were fixed, stained with sulphorhodamine B, absorbances measured at 560 nm and cell survivals were determined. Cytotoxicity assays were also performed with doxorubicin (Sigma Chemical). The cytotoxicity curves for Pac-10 cells with and without verapamil were similar for paclitaxel. Pac-10 cells selected in the absence of verapamil were negative for MDR-1, as shown below. Therefore, for all of the experiments described below, Pac-10 cells selected in the absence of verapamil were used. Cytotoxicity assays described above were also performed by using Pac 2, 4, 6 and 8 cell lines to determine the resistance to paclitaxel.

### Drug accumulation and efflux assay

[$^3$H]taxol was purchased from Movarek Biochemicals. DU-145 and Pac-10 were plated onto 24-well plates and grown to approximately 90% confluency. Cells were rinsed with PBS and incubated in medium containing 0.1 μCi $ml^{-1}$ [$^3$H]taxol (spec. act. 11.6 Ci $mmol^{-1}$) for 60 min at 37°C. At the end of incubation, cells were washed thoroughly and intracellular $^3$H drug was determined by scintillation counting of solubilized cells. This initial solubilization was taken as zero point and used to determine the accumulation of the drug. Samples were collected at 45 and 90 min after the zero point to determine the efflux (Zilfou and Smith, 1995).

### Flow cytometric cell cycle analysis

This was performed by standard methodology after propidium iodide staining of cellular DNA content. The percentage of cells in $G_1$, S and $G_2$-M phases was calculated for DU-145 and Pac-10 cells (Vindelov et al, 1983).

### β-Tubulin isotype staining by immunofluorescence

DU-145 cells and Pac-10 cells were plated on glass coverslips and stained with β-tubulin antibodies, as described previously (Ranganathan et al, 1996). $\beta_{II}$-, $\beta_{III}$- and $\beta_{IV}$-antibodies were purchased from Biogenex Laboratories (San Ramon, CA, USA). General α-tubulin and β-tubulin antibodies were purchased from Sigma Chemical and used at 1:200 dilution. Texas-red conjugated anti-mouse IgG (Molecular Probes, Eugene, OR, USA) was used as a secondary antibody at 1:200 dilution. Stained cells were scanned using the confocal microscopy system.

### Protein analysis using western blots

Crude cytosolic lysates were prepared from DU-145 and Pac-10 cells, protein concentrations were estimated, run on 8% polyacrylamide gels and transferred onto PVDF membranes, as described previously (Ranganathan et al, 1996). Blots were stained with isotype-specific and non-specific β-tubulin antibodies. Similar analysis was performed using Pac 2, 4, 6 and 8 cell lines with $\beta_{III}$-antibodies.

### RT-PCR analysis of β-tubulin isotype transcripts and MDR-1

RNA was isolated from DU-145 and Pac-10 cell lines by a modified acid–guanidium lysis procedure (Chomczynski and Sacchi 1987). Primers were chosen using the Primer Detective Program (Clontech, Palo Alto, CA, USA) and were synthesized by the DNA core facility at Fox Chase Cancer Center. β-Tubulin isotype-specific primers for the $\beta_I$-, $\beta_{II}$-, $\beta_{III}$- and $\beta_{IV}$-isotypes were described in detail previously (Ranganathan et al, 1996). The MDR-1 primers (nucleotides 1325–1347, 1502–1523 of the cDNA sequence) are specific for MDR-1 and span an intron to differentiate between amplified products derived from RNA and DNA. PCR products were analysed using agarose gel electrophoresis and quantified by scanning densitometry.

### RESULTS

### Resistance to paclitaxel and response to other anti-microtubule agents

As shown in Figure 1A, Pac-10 cells were fivefold resistant to paclitaxel ($IC_{50}$ 10 nM) compared with the DU-145 cells ($IC_{50}$ 2 nM). Pac-10 cells were only twofold cross-resistant to estramustine, with $IC_{50}$ values of 2.5 μM for DU-145 cells and 5 μM for the Pac-10 cells. Similarly, twofold cross-resistance to colchicine was seen with $IC_{50}$ values of 50 and 100 nM for DU-145 and Pac-10 cells respectively. The cytotoxicity curves for vinblastine were similar for both cell lines with an $IC_{50}$ value of 2 nM (data not shown).

### Paclitaxel resistance is not MDR-1 mediated

RT-PCR analysis of DU-145 cells and Pac-10 cells showed that both cell lines were negative for MDR-1 (Figure 1B). In addition to this, drug accumulation and efflux experiments using [$^3$H]taxol have shown that Pac-10 cells were very similar to DU-145 cells in taxol accumulation. Efflux rates were also similar, with 50% of the drug being eliminated from cells by 90 min (Table 1). Thus, the resistance of Pac-10 cells is not due to decreased paclitaxel entry or increased efflux from the cells. Pac-10 cells were only twofold resistant to doxorubicin compared with DU 145 cells, with $IC_{50}$ values of 200 and 100 nM respectively. This cross-resistance to doxorubicin, estramustine and colchicine was probably due to the slower growth rate of Pac-10 cells. Cell doubling times for DU 145 and Pac-10 cells were approximately 18 h and 30 h respectively.

### Cell cycle analysis

Paclitaxel is known to block cell cycle progression in late $G_2$-M phases. To examine its effects on Pac-10 cell cycle, DNA was stained by propidium iodide and analysed by FACS. The results showed that there were no alterations in the percentage of cells in $G_1$, S- or $G_2$-M phases in paclitaxel-resistant cells compared with the DU-145 cells (data not shown).

### β-Tubulin isotype analysis by immunofluorescence and western blots

When the DU-145 and Pac-10 cells were stained with isotype non-specific tubulin antibodies and examined by confocal microscopy,

© Cancer Research Campaign 1998

**Figure 1** (**A**) Cytotoxicity curves for paclitaxel. Drug-resistant cells and parental DU-145 cells were treated with various concentrations of paclitaxel for 48 h and cytotoxicities were determined as described in Materials and methods. Data shown are means, bars = s.d. (**B**) MDR-1 analysis by RT-PCR in DU-145 and paclitaxel resistant cells. RNA was isolated from both cell lines and MDR-1 transcript was amplified by using MDR-1-specific primers. RT-PCR products were analysed using agarose gel electrophoresis. KB8-5, an MDR-1 expressing cell line, was used as a positive control increased levels of total α- and β-tubulin protein were observed in the resistant cells (Figure 2). This was confirmed by western blot analysis and the increase for total α- and β-tubulin levels was approximately threefold (data not shown). The $\beta_{II}$- and $\beta_{IV}$-isotypes were present at low levels in both cell lines. $\beta_{III}$-Isotype levels increased significantly in Pac-10 cells when compared with the parental DU-145 cells (Figure 2). This increase of $\beta_{III}$ was confirmed by Western blot (see Figure 4) and quantitated to be approximately fourfold elevated.

### Analysis of β-tubulin isotypes by RT-PCR

Total RNA isolated from DU-145 and Pac-10 cells was used to amplify the β-tubulin isotype transcripts and the PCR products were analysed by agarose gel electrophoresis. The results were quantitated by using 18S RNA for normalization as described previously (Ranganathan et al, 1996) (Figure 3). These data show that the $\beta_{III}$-transcript levels were ninefold higher in Pac-10 cells compared with DU-145 cells. In addition, there was a fivefold increase of $\beta_{IVa}$-isotype in the resistant cells. This increase of $\beta_{IVa}$-isotype could not be confirmed by western blot analysis because of lack of $\beta_{IVa}$-specific antibodies. $\beta_{I}$- and $\beta_{IVb}$-transcripts were the predominant isotypes in both cell lines and did not appear to change in Pac-10 cells as a result of resistance. The $\beta_{II}$-isotype could not be detected in either of the cell lines under the PCR conditions (26 cycles). At 30 cycles, a faint band was seen with equal intensity in both cell lines (data not shown).

**Table 1** Efflux of [$^3$H]taxol from DU145 and Pac10 cells. Cells were incubated with [$^3$H]taxol for 60 min at 37°C. Cells were washed in PBS, solubilized and accumulated [$^3$H]taxol in cells and efflux of the drug from cells was measured. Values represent means ±s.d. of three separate experiments

| Time | DU145 | Pac-10 |
|---|---|---|
| 0 | 100% | 100% |
| 45 | 73.7 ± 6.5 | 67.6 ± 6.6 |
| 90 | 51.4 ± 4.9 | 45.1 ± 12.0 |

### Correlation between paclitaxel resistance and $\beta_{III}$-levels

To investigate further the relationship between paclitaxel resistance and the expression of $\beta_{III}$-isotype, cells that were selected at different concentrations of the drug were analysed as shown in Figure 4. Exposure to increasing levels of paclitaxel resulted in a gradient of increasing resistance to the drug. Western blot analysis of the cytosolic proteins from these cell lines showed a similar gradient of increasing $\beta_{III}$-isotype protein concentrations from Pac-2 to Pac-10 cells (Figure 4, inset).

### DISCUSSION

The $\beta_{III}$-isotype differs significantly from the other β-tubulin isotypes in its C-terminal amino acid composition (Sullivan, 1988). Therefore, it may differ from the other isotypes in its microtubule assembly properties. Indeed, experiments performed with bovine brain tubulin in the presence of microtubule associated proteins (MAPs) have shown that the $\beta_{III}$-depleted tubulin polymerizes at a faster rate than unfractionated tubulin (Banerjee et al, 1990). Further study from the same laboratory has shown that the $\beta_{III}$-depleted tubulin was also more sensitive to paclitaxel-induced polymerization compared with unfractionated tubulin (Lu and Luduena, 1993). The critical concentration of tubulin required for microtubule assembly in the presence of paclitaxel was approximately three times greater for the unfractionated bovine brain tubulin compared with the $\beta_{III}$-depleted tubulin. In addition microtubules assembled from the $\beta_{III}$-depleted tubulin were shorter and more resistant to podophyllotoxin and colchine compared with the microtubules from unfractionated tubulin.

In earlier studies from our laboratory, we had observed increases of the $\beta_{III}$- and $\beta_{IVa}$-tubulin isotypes in human prostate carcinoma cells that had been made resistant to estramustine, a microtubule-depolymerizing agent (Ranganathan et al, 1996). These cells were also partially cross-resistant to paclitaxel. In this study, we observed an increase in $\beta_{III}$- and $\beta_{IVa}$-levels in paclitaxel resistant cells, similar

© Cancer Research Campaign 1998

**Figure 2** Immunofluorescent staining of parental and drug-resistant cells with α- and β-tubulin antibodies and β-tubulin isotype-specific antibodies. Cells were plated on coverslips, fixed and stained with the antibodies as described in Materials and methods

**Figure 3** Quantification of the RT-PCR analyses of the β-tubulin isotype transcripts for the drug-resistant and parental cell lines. Experiments were conducted at least six times and the relative abundance of transcripts was calculated as a ratio of 18S to the isotype. ░, DU145; ■, PACI0

**Figure 4** Correlation between paclitaxel resistance and $\beta_{III}$ tubulin levels. Pac 2, 4, 6, 8 and 10 cell lines were treated with paclitaxel for 48 h and compared with DU-145 cells to determine the level of resistance. Same cell lines were also analysed for the $\beta_{III}$ levels by western blot analysis as described in the Materials and methods to the increases seen in estramustine-resistant cells. The positive correlation between the extent of paclitaxel resistance and the concentration of $\beta_{III}$-isotype indicates that increased expression of $\beta_{III}$-tubulin may play a significant role in resistance to the drug.

Previous studies by other groups have shown that anti-microtubule agents such as paclitaxel, vinblastine and colchicine act by suppressing microtubule dynamics in vitro and in cells (Jordan et al, 1992; Derry et al, 1995; Dhamodharan et al, 1995). Using purified β-tubulin isotypes, Panda et al (1994) have shown that microtubule dynamics are regulated by the tubulin isotype composition. Microtubules assembled from purified $\alpha\beta_{III}$-isotype were more dynamic than microtubules made from $\alpha\beta_{II}$- and $\alpha\beta_{IV}$-isotypes or unfractionated tubulin. Moreover, microtubules composed of $\alpha\beta_{III}$- and $\alpha\beta_{IV}$-isotypes were four times less sensitive to inhibition of microtubule dynamics by paclitaxel (Derry et al, 1997). Based on these studies, it is reasonable to speculate that the increases of $\beta_{III}$ and $\beta_{IVa}$ seen in our drug-resistant cell lines may alter the microtubule dynamics in these cells to overcome the effects of anti-microtubule agents. An additional explanation for the increases in the isotypes may be altered drug-binding. This hypothesis is supported by less efficient binding of [$^{14}$C]estramustine into $\beta_{III}$-isotype, compared with other isotypes (Laing et al, 1997). The drug-binding sites for paclitaxel, however, appear to be in the areas of high homology among all of the isotypes.

Haber et al (1995) have shown overexpression of $\beta_{II}$-isotype in a paclitaxel-resistant murine cell line that also overexpresses P-glycoprotein. In contrast, the paclitaxel-resistant DU-145 cells described in our study do not overexpress the MDR-1 gene product. Alterations in β-tubulin isotypes due to paclitaxel resistance have also been reported in other cell lines such as sarcoma, breast carcinoma and ovarian carcinoma (Giannakakou et al, 1996; Mallarino et al, 1996). These studies indicate that the drug-induced change in isotype composition may be a general mechanism of resistance to paclitaxel and other agents that perturb microtubule dynamics.

Previously, we hypothesized that the favourable interaction of paclitaxel and estramustine in the treatment of patients with hormone refractory prostate cancer was based on the complementary but different targets within the microtubule, i.e. paclitaxel binding to β-tubulin and estramustine binding to microtubule-associated proteins and tubulin (Dahllof et al, 1993; Speicher et al, 1994; Laing et al, 1997). The present results and our previous findings in estramustine-resistant prostate cell lines suggest that resistance to paclitaxel and estramustine may share a common basis despite the differing binding sites on the microtubule. Regardless of how an agent interacts with the microtubule, the common effect of decreasing microtubule dynamics may explain the similar alteration in β-tubulin isotype pattern in estramustine- and paclitaxel-resistant cell lines. Increasing microtubule dynamics by altering tubulin isotypes may constitute a general mechanism by which the cell can defend itself against the large number of natural compounds which inhibit microtubule dynamics. To further understand the significance of the $\beta_{III}$- and $\beta_{IV}$-isotypes in anti-microtubule drug therapy, transfection experiments using the $\beta_{III}$ and $\beta_{IVa}$ cDNAs are underway.

As single agents, estramustine (Hudes, 1997) and paclitaxel (Roth, 1993) are inactive in treatment of hormone refractory prostate cancer but given together these drugs show significant activity in preclinical (Speicher et al, 1992) and clinical studies (Hudes et al, 1995). The mechanisms for greater than additive preclinical and clinical anti-tumour activity of paclitaxel/estramustine combination remains to be elucidated. Binding of one of these agents to the microtubules may alter the conformation of the target, thus facilitating binding of the second agent. The C-terminus of β-tubulin peptide has been shown to be important in MAP binding and folding of the molecule (Cross et al, 1994; Fontalba et al, 1995). Differing significantly from the other isotypes in its C terminus, the $\beta_{III}$-isotope may also have distinct MAP-binding properties. Thus, it may be important for the synergistic activity of the paclitaxel/estramustine combination.

### ACKNOWLEDGEMENTS

This work was supported by Public Health Service Grants CA-57638 and CA-06927 from the National Cancer Institute and a grant from AHEPA 5th District Cancer Research Foundation. PJC is a recipient of clinical investigator training grant. We thank Anne Carson for typing the manuscript.

### REFERENCES

Banerjee A, Roach MC, Trcka P and Luduena RF (1990) Increased microtubule assembly in bovine brain tubulin lacking type III isotype of β-tubulin. *J Biol Chem* **265**: 1794–1799

Chomczynski P and Sacchi N (1987) Single-step method of RNA isolation by acid guanidinium thiocyanate-phenol-chloroform extraction. *Anal Biochem* **162**: 156–159

Cross D, Gustavo F, Dominguez J, Avila J and Maccioni RB (1994) Carboxyl terminal sequences of β-tubulin involved in the interaction of HMW-MAPs. Studies using site-specific antibodies. *Mol Cell Biochem* **32**: 81–90

Dahllof B, Billstrom A, Cabral F and Hartley-Asp B (1993) Estramustine depolymerizes microtubules by binding to tubulin. *Cancer Res* **53**: 4573–4581

Derry WB, Wilson L and Jordan MA (1995) Substoichiometric binding of taxol suppresses microtubule dynamics. *Biochemistry* **34**: 2203–2211

Derry WB, Wilson L, Khan IA, Luduena FR and Jordan MA (1997) Taxol differentially modulates the dynamics of microtubules assembled from unfractionated and purified β-tubulin isotypes. *Biochemistry* **36**: 3554–3562

Dhamodharan R, Jordan MA, Thrower D, Wilson LW and Wadsworth P (1995) Vinblastine suppresses dynamics of individual microtubules in living interphase cells. *Mol Biol Cell* **6**: 1215–1229

Fontalba A, Avila J and Zabala JC (1995) β-tubulin folding is modulated by the isotype-specific carboxy-terminal domain. *J Mol Biol* **246**: 628–636

Giannakakou P, Sackett D, Kang YK, Fojo AT and Poruchynsky M (1996) Tubulin from paclitaxel (PTX) resistant human ovarian carcinoma cell lines demonstrates altered response to drug, *in vivo* and *in vitro* (abstract 2176). *AACR 87th Annual Meeting*, Washington DC, Vol. 37

Haber M, Burkhart CA, Regl DL, Madafiglio J, Norris MD and Horwitz SB (1995) Altered expression of Mβ2, the class II β-tubulin isotype, in a murine J774.2 cell line with a high level of taxol resistance. *J Biol Chem* **270**: 31269–31275

Horwitz SB, Rao S, Haber M, Burkhart C and Orr GA (1995) The taxol-binding site on the microtubule (abstract). *Cytoskeleton and Cancer: Proceedings* 42

Hudes GR, Nathan FE, Khater C, Haas N, Cornfield M, Giantonio B, Greenberg R, Gomella L, Litwins S, Ross E, Roetmke S and McAleer C (1997) Phase II trial of 96-hour paclitaxel plus oral estramustine phosphate in metastatic hormone – refractory prostate cancer. *J Clin Oncol* **15**: 3156–3163

Hudes GR (1997) Estramustine-based chemotherapy. *Sem Urol Oncol* **15**: 13–19

Jordan MA, Thrower D and Wilson L (1992) Effects of vinblastine, podophyllotoxin and nocodazole on mitotic spindles: implications for the role of microtubule dynamics in mitosis. *J Cell Sci* **102**: 401–416

Jordan MA, Toso RJ, Thrower D and Wilson L (1993) Mechanism of mitotic block and inhibition of cell proliferation by taxol at low concentrations. *Proc Natl Acad Sci USA* **90**: 9552–9556

Laing N, Dahllof B, Hartley-Asp B, Ranganathan S and Tew K (1997) Interaction of estramustine with tubulin isotypes. *Biochemistry* **36**: 871–878

Lu Q and Luduena RF (1993) Removal of $\beta_{III}$ isotypes enhances taxol induced microtubule assembly. *Cell Struct Funct* **18**: 173–182

Mallarino MC, Duran GE, Dumontet C, Cluck M, Mangili A and Sikic BI (1996) The spectrum of resistance to paclitaxel in human sarcoma and breast cancer cells selected with and without the multidrug resistance (MDR-1) modulator PSC 833 (abstract 2987). *AACR 87th Annual Meeting*, Washington DC, Vol. 37

Panda D, Miller HP, Banerjee A, Luduena RF and Wilson L (1994) Microtubule dynamics *in vitro* are regulated by the tubulin isotype composition. *Proc Natl Acad Sci USA* **91**: 11358–11362

Ranganathan S, Dexter DW, Benetatos CA, Chapman AE, Tew KD and Hudes GR (1996) Increase of $\beta_{III}$- and $\beta_{IVa}$-tubulin isotypes in human prostate carcinoma cells as a result of estramustine resistance. *Cancer Res* **56**: 2584–2589

Roth BJ, Yeap BY, Wilding G, Warren J-T, Bokesch H, Kenny S and Boyd MR (1993) Taxol in advanced, hormone-refractory carcinoma of the prostate. A phase II trial of the Eastern Cooperative Oncology Group. *Cancer* **72**: 2457–2460

Skehan P, Stoneng R, Scudiero D, Monks A, McMahon J, Vistica D, Kasimis B, McLeod D and Loehler PS (1990) New colorimetric cytotoxicity assay for anticancer-drug screening. *J Natl Cancer Inst* **82**: 1107–1112

Speicher LA, Barone L and Tew K (1992) Combined antimicrotubule activity of estramustine and taxol in human prostatic carcinoma cell lines. *Cancer Res* **52**: 4433–4440

Speicher LA, Laing N, Barone LR, Robbins JD, Seamon KB and Tew KD (1994) Interaction of an estramustine photoaffinity analogue with cytoskeletal proteins in prostate carcinoma cells. *Mol Pharmacol* **46**: 866–872

Sullivan KF (1988) Structure and utilization of tubulin isotypes. *Annu Rev Cell Biol* **4**: 687–716

Vindelov LL, Christensen IJ and Nissen NI (1983) A detergent-trypsin method for the preparation of nuclei for flow cytometric DNA analysis. *Cytometry* **3**: 323

Zilfou JT and Smith CD (1995) Differential interactions of cytochalasins with P-glycoprotein. *Oncol Res* **7**: 435–443

© Cancer Research Campaign 1998